# Management of labyrinthine fistula: hearing preservation versus prevention of residual disease

**DOI:** 10.1007/s00405-017-4697-2

**Published:** 2017-08-10

**Authors:** S. Geerse, M. J. F. de Wolf, F. A. Ebbens, E. van Spronsen

**Affiliations:** Department of Otorhinolaryngology Head and Neck Surgery, Academic Medical Center, Meibergdreef 9, 1105 AZ Amsterdam, The Netherlands

**Keywords:** Labyrinthine fistula, Cholesteatoma, Computed tomography, Ear surgery, Bone conduction threshold

## Abstract

The objective of the study was to evaluate postoperative hearing and disease control after cholesteatoma surgery for labyrinthine fistulas. In a retrospective cohort study, we evaluated a consecutive cohort comprising 44 patients (45 ears) with labyrinthine fistulas associated with chronic otitis media with cholesteatoma who underwent surgery between 2002 and 2015. We looked at patient characteristics, pre- and postoperative bone conduction thresholds (BCT), operative approach and findings, extent of disease and the occurrence of residual disease. All deaf ears (24%) presented preoperatively with a large fistula. Opening the membranous labyrinth resulted in significantly worse postoperative BCT (*p* = 0.01). Neither the present study nor a literature search revealed a significant positive effect of corticosteroids on postoperative hearing preservation. Large fistulas were correlated with poorer preoperative BCTs, but not with poorer postoperative BCTs. Opening the membranous labyrinth during surgery is correlated with poorer postoperative BCTs and can be seen as a predictive parameter. The use of corticosteroids in the perioperative management of labyrinthine fistula was not found to result in any improvement in postoperative BCTs.

## Introduction

Labyrinthine fistulas are a well-known complication of chronic otitis media with cholesteatoma. Reported incidences vary from 4 to 15% [1–5]. As the labyrinth is connected to the cochlea, a labyrinthine fistula can lead to sensorineural hearing loss in addition to vertigo [6]. Surgery is challenging due to the increased risk of iatrogenic sensorineural hearing loss. Several classifications have been proposed for fistula size. However, there has not yet been any assessment of the prognostic value for postoperative hearing preservation [2, 7, 8]. At present, these classifications can only be used as a general description of size. It would be very useful to have clinically relevant prognostic parameters that can predict the chances of preserving hearing and labyrinthine function. Parameters of this kind could improve our preoperative counselling. Several surgical techniques have been proposed for optimising the exposure of the fistula to allow meticulous eradication of the cholesteatoma [5]. At present, the optimal surgical management of these fistulas is a topic of debate [1, 9].

Positive effects on postoperative hearing have been described when intravenous corticosteroids are applied during surgery [3, 10, 11]. Obliteration of the mastoid is thought to reduce postoperative dizziness [10]. The management of cholesteatoma-induced labyrinthine fistulas consists of the complete removal of the cholesteatoma matrix from the fistula and the prevention of sensorineural hearing loss and dizziness postoperatively due to iatrogenic damage. The first objective of this study was to evaluate the postoperative hearing results in relation to several prognostic variables such as type of surgery, the size of the fistula and the extent of affected inner ear structures. The second objective was to evaluate disease control after cholesteatoma surgery when a labyrinthine fistula is present. Jang et al. had favourable results in their cohort with the use of perioperative intravenous corticosteroids [10]. We will compare their results with ours (without the use of corticosteroids) and present an overview of the literature regarding this aspect of labyrinthine management.

## Patients and methods

### Patients

**Fig. 1:**
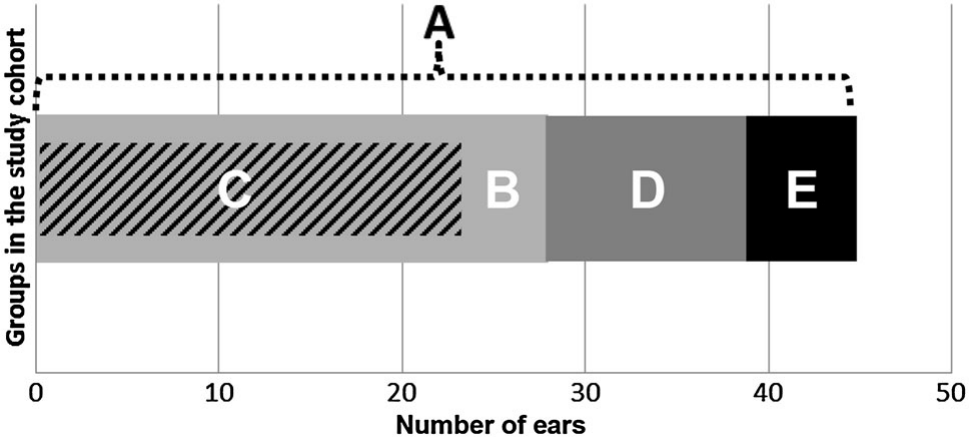
Subgroups in the study cohort. *A* Total cohort, *B* evaluation of postoperative hearing with complete hearing tests, *C* evaluation of postoperative hearing with complete hearing tests and only fistula in LSC, *D* deaf ears, *E* ears without complete hearing tests

A retrospective chart review was conducted of mastoid surgery for cholesteatoma performed at the Department of Otolaryngology of the Academic Medical Centre between 2002 and 2015. All patients with labyrinthine fistulas associated with cholesteatoma were selected. The study cohort was divided into several subgroups (Fig. 1). The total cohort was defined as group A for the purposes of determining the occurrence of postoperative disease. To determine the difference between pre- and postoperative hearing, a selection was made of the patients with functional hearing preoperatively and adequate hearing tests with bone conduction thresholds (BCT) at 1, 2 and 4 kHz pre- and postoperatively (group B). Group C consisted of the patients from group B who had a fistula in the lateral semicircular canal (LSC) only. This group was established to compare our hearing results with those of Jang et al. [10], who used intravenous corticosteroids intraoperatively. Deaf ears preoperatively (group D) or patients with incomplete hearing tests (group E) were not used for comparison of pre- and postoperative hearing.

### Surgical technique

Several surgical techniques were used in our study population. Until about 2007, the usual approach in our centre was to perform canal wall down mastoidectomy (CWDM) or revision radical cavity surgery (RRCS) when a fistula was present. After 2007, our philosophy shifted towards canal wall up mastoidectomy (CWUM), which is also referred to as combined approach tympanoplasty (CAT), with second look or obliteration. Alternatively, in cases with radical cavities, we used RRCS with partial hydroxy-apatite obliteration and reconstruction of the canal wall (PHORC). Subtotal petrosectomies (STP) were performed when there was a deaf ear preoperatively. The technique adopted depended on previous surgery, preoperative hearing status and the extent of the disease, and not on the presence of one or more fistulas. In all cases, the cholesteatoma was completely removed and the fistula was covered with fascia and fibrin glue. Mastoid obliteration was performed in some patients in the cohort as the preference for this technique increased. This did not depend on the presence of any fistula. No intravenous corticosteroids were used before or during surgery.

### Fistulas

We evaluated the extent of the damage to the labyrinth and the extent of the involvement of the inner ear structures. Six inner ear structures were defined: lateral semicircular canal (LSC), superior semicircular canal (SSC), posterior semicircular canal (PSC), cochlea, utricle and saccule. We used the classification proposed by Sanna et al. [7] to grade the size of the fistula. This classification defines three types of fistula: small fistulas (0.5–1 mm), medium (1–2 mm) and large fistulas (>2 mm). Sanna et al. [7] measured the fistulas intraoperatively. In the current cohort, radiological images were used to grade fistula size. Preoperative high-resolution computed tomography (HRCT) scan images with 0.6 mm thickness were obtained routinely for all patients using a Philips Brilliance 64 CT Scanner. A radiologist measured all fistulas in the axial plane using the technique described by Jang et al. [10] and Sone et al. [12] on the outer surface of the defect.

### Hearing

Preoperative and postoperative pure-tone averages were measured for the bone conduction threshold (BCT) at 1, 2 and 4 kHz with a clinical audiometer calibrated according to ISO standards to assess sensorineural hearing thresholds. We defined significant sensorineural hearing loss as >10 dB loss at two or more frequencies. Hearing improvement was defined as 10 dB or more improvement at two or more frequencies [10, 11]. The following parameters were evaluated to determine the possible effect on pre- or postoperative hearing: fistula size, primary or revision surgery, surgical technique, opening of the membranous labyrinth intraoperatively, extent of affected inner ear structures and the specific structure involved. Also, the use of perioperative corticosteroids was evaluated comparing our data to those of Jang et al. [10]. A non-systematic literature search was performed using PubMed and Medline databases and the following MeSH terms (Cholesteatoma) and (Fistula) to evaluate postoperative BCT with and without the use of intravenous corticosteroids during surgery. Publications were included if they specifically described whether they used corticosteroids intraoperatively or not for the management of labyrinthine fistula.

### Disease control

Otoscopic follow-up and MRI-DWI (diffusion weighted magnetic resonance imaging) 1 year postoperatively were used to determine whether there was any residual or recurrent cholesteatoma. We looked at group A to determine whether there was any residual cholesteatoma in the region of the former fistula or in the obliterated cavity. Given the importance of striking a balance between disease control and hearing preservation, postoperative BCT was evaluated with and without residual cholesteatoma. Postoperative dizziness was evaluated in relation to obliteration of the mastoid. The need for revision surgery was also evaluated.

### Statistical analysis

Microsoft Excel 2010 was used for statistical analysis. Linear trend analysis was performed on hearing results to our data with those of Jang et al. [10]. Patient age and time of follow-up were stated as numbers and medians (range). Fisher’s exact test was performed on the different variables related to postoperative hearing, postoperative dizziness in relation to obliteration and on the presence of residual cholesteatoma and postoperative BCT.

## Results

### Patients

A total of 690 mastoid surgery cases for cholesteatoma were identified between 2002 and 2015. Seven percent of these cases—a total of 44 patients with 45 ears—were identified as having labyrinthine fistulas (group A) by evaluating all surgery reports. Twenty-three ears in these labyrinthine fistula cases (51%) were revision surgery cases. The study cohort consisted of 19 females and 25 males with a median age of 49 years (range 16–76). Follow-up time ranged from 2 to 157 months with a median of 30 months. Twenty-eight ears were selected to evaluate postoperative hearing results (group B) by excluding the cases with preoperative deaf ears (*n* = 11) and those with incomplete audiometry (*n* = 6).

### Surgical technique

Several surgical techniques were used in our cohort. A radical cavity was created in four cases (9% of the total cohort). A PHORC procedure was performed in 10 cases (22%) and 19 cases (42%) underwent a CAT procedure. An STP was performed in 12 cases (27%). Obliteration of the mastoid was performed in 29 cases (64%), 21 of which (47%) had revision surgery after CWDM. The membranous labyrinth was inadvertently opened during surgery in 20% of the total cohort. Suction was never applied directly to the fistulas.

### Fistulas

Preoperative HRCT scan evaluation revealed 100% of the fistulas in the current cohort, making it a very specific diagnostic modality. The evaluation of which specific structures were involved was done by HRCT and through evaluation of surgical reports. The LSC only was involved in 73% of the cases (33/45) (group C). A fistula in the LSC combined with other inner ear structures was found in 20% (9/45). The LSC was not involved at all in only 7% of the cases (3/45). The cochlea was involved in 11% (5/45). Up to six structures were involved in one case. Sixty percent of the fistulas were graded as large using the Sanna classification [7] (Table 1).

**Table 1:** Patient characteristics

	Fistula size graded using Sanna classification^1^			Preop dizziness	Preop deaf ears	Primary surgery	Revision surgery
	Small (0.5–1 mm)	Medium (1–2 mm)	Large (>2 mm)				
Female	1	6	12	14	7	8	11
Male	4	7	15	14	4	14	12
Total (*n* = 45)	5 (11%)	13 (29%)	27 (60%)	28 (62%)	11 (24%)	22 (49%)	23 (51%)

### Preoperative hearing

In group A, the majority (47%) of the cases had a bone conduction threshold of 11–20 dB (Table 2). In the total cohort of 45 cases, 11 ears (24%) were deaf prior to surgery (group D). None of these cases involved ears with fistulas graded as small. Ten out of 11 cases (91%) in group D had large fistulas.

**Table 2:** Average preoperative bone conduction threshold (1, 2 and 4 kHz) and Sanna classification^1^

	Small (0.5–1 mm)	Medium (1–2 mm)	Large (>2 mm)	Total
0–10	1	1	4	6
11–20	3	7	6	16
21–30	1	4	2	7
31–40	0	0	1	1
41–50	0	0	2	2
51–60	0	0	1	1
61–70	0	0	0	0
71–80	0	0	1	1
81–90	0	0	0	0
Deaf	0	1	10	11
Total	5	13	27	45

### Parameters with possible prognostic value for postoperative hearing

**Fig. 2:**
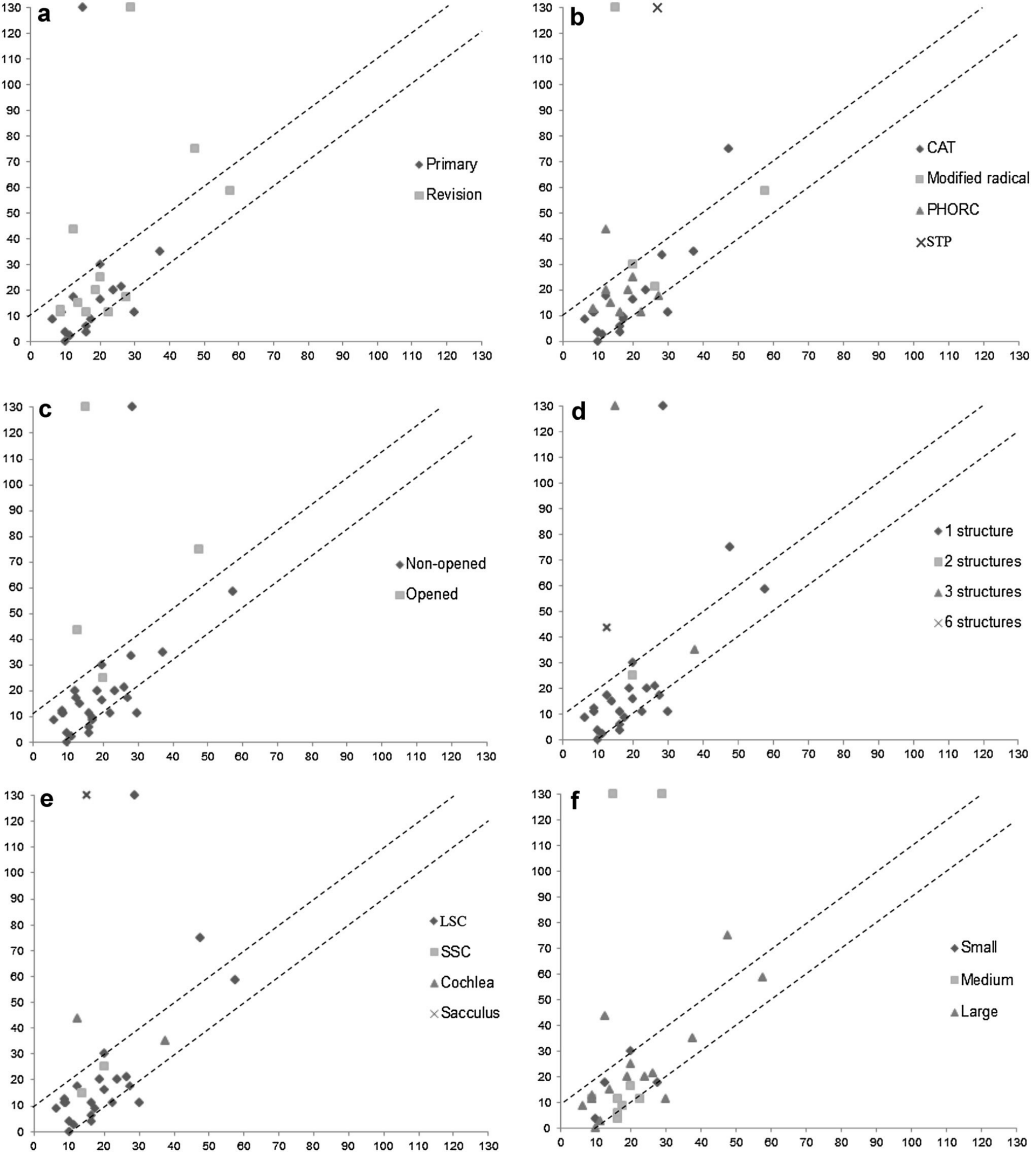
Pre- (*x-axis*) and postoperative (*y-axis*) hearing (PTA in dB) in group B as related to different variables. **a** Primary or revision surgery. **b** Type of surgery (*CAT* combined approach tympanoplasty, *PHORC* partial hydroxy-apatite obliteration and reconstruction of the canal wall, *STP* subtotal petrosectomy). **c** Membranous labyrinth opened or not. **d** Number of damaged labyrinthine structures. **e** Specific labyrinthine structures (*LSC* lateral semicircular canal, *SSC* superior semicircular canal). **f** Fistula size graded with the Sanna classification^1^

In 24 cases (86%) in group B, postoperative hearing was improved or unchanged; in 2 cases (7%) hearing deteriorated and 2 deaf ears (7%) were found postoperatively. We have described group B in Fig. 2 using several prognostic parameters. We found that 25% of the ears with deteriorated hearing and deafness had undergone primary surgery and that 75% were revision cases (Fig. 2a). In the cases where hearing was not affected, 63% had undergone primary surgery and 37% were revision cases. Fisher’s exact test showed no statistical significance in the distribution of these cases (*p* value = 0.34). As can be seen in Fig. 2b, the various surgical techniques that were used all had a case in which deteriorated BCT was found postoperatively. The percentages were 6% in CAT, 25% in CWD, 11% in PHORC and 100% in STP. The last subgroup consisted of one case.

**Fig. 3:**
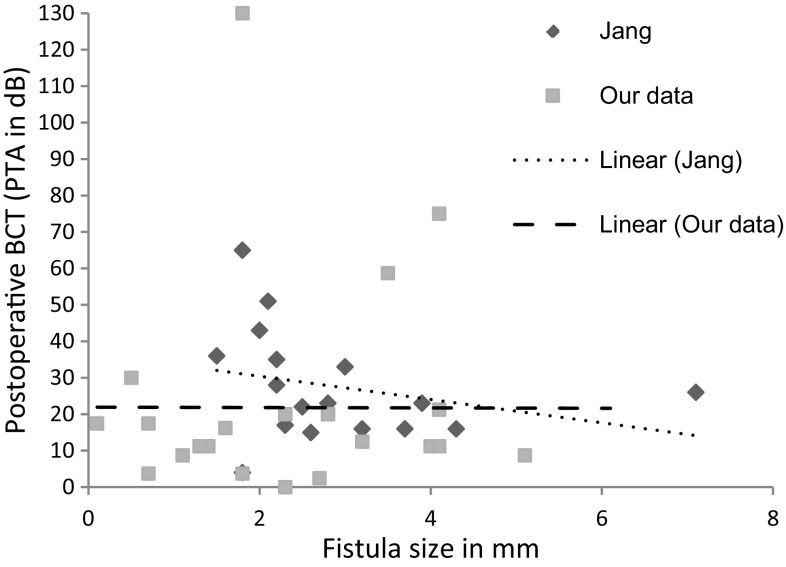
Comparison of our data with the data of Jang et al.^1^ for postoperative hearing (PTA in dB) in relation to fistula size (mm)

Group B consisted of four cases in which the membranous labyrinth was opened during surgery. Three of these cases (75%) had deteriorated hearing after surgery, one of which had a deaf ear postoperatively (Fig. 2c). This rate of deaf ears was much higher than in cases in which the membranous labyrinth was not opened (1 in 20: 5%). Fisher’s exact test showed that this difference in this distribution between the groups was significant (*p* value = 0.01). The number of labyrinthine structures and the specific structures damaged had no prognostic value (*p* value >0.05) (Fig. 2d, e), although there seems to be a trend for these parameters (*p* value <0.1) towards a negative prognosis if more than one structure was affected. Figure 2F shows that medium and large fistulas can lead to both deteriorated and improved hearing (*p* value >0.05). Postoperative hearing in group C was compared with the data of Jang et al. [10] to evaluate the effect of corticosteroids intraoperatively. Figure 3 shows the trend lines for the two studies: there is no significant difference between the data. The literature search did not reveal any differences between the studies which used corticosteroids and those which did not (Table 3). The mean hearing results in the corticosteroid studies were 90% unchanged or improved, 5% deteriorated and 5% deaf ears. The studies which did not use corticosteroids had a mean of 93% improved or unchanged hearing, 5% deteriorated and 2% deaf ears. When these data were analysed, no significant differences were found between the groups in the distribution of deaf ears or ears with deteriorated hearing (*p* value >0.5).

**Table 3:** Postoperative hearing results with or without use of corticosteroids

Author	Year	Corticosteroids used	Hearing improved or unchanged	Hearing deteriorated	Deaf ear
Dornhoffer	1995	Yes	13 (93%)	1 (7%)	0 (0%)
Gocea	2011	Yes	22 (85%)	4 (15%)	0 (0%)
Meyer	2015	Yes	4 (80%)	0 (0%)	1 (20%)
Jang	2015	Yes	17 (100%)	0 (0%)	0 (0%)
		Mean	90%	5%	5%
Quaranta	2009	No	43 (96%)	0 (0%)	2 (4%)
Ueda	2009	No	24 (89%)	3 (11%)	0 (0%)
Moon	2011	No	26 (93%)	2 (7%)	0 (0%)
Stephenson	2011	No	28 (100%)	0 (0%)	0 (0%)
Ikeda	2012	No	35 (92%)	3 (8%)	0 (0%)
Geerse	2017	No	24 (86%)	2 (7%)	2 (7%)
		Mean	93%	5%	2%

### Disease control

#### Recurrences and residual disease

Six recurrent cholesteatomas (14%) were found by otoscopic evaluation with a median follow-up time of 18 months (range 10–22). A residual cholesteatoma was found in four cases (9%) using MRI-DWI. Two residual cholesteatomas were found on the dehiscent facial nerve (after 12 and 14 months), one on the carotid artery (after 12 months) and one in the supralabyrinthine area (after 35 months). No residual cholesteatomas were found on the labyrinthine fistula or in cases where obliteration of the mastoid was performed. Mean BCT in the residual group improved from 16.58 dB preoperatively to 12.50 dB postoperatively. In the group with no residual cholesteatomas, the mean BCT worsened from 20.58 dB to 28.54 dB postoperatively. However, this difference was not significant (*p* = 0.13).

#### Control of presenting complaints

Twenty-eight patients (62%) presented with vertigo complaints preoperatively. Postoperatively, ten patients (22%) had some dizziness lasting between a few days and a few weeks. Nine of these patients had preoperative dizziness as well. No difference was found in postoperative dizziness between the group with mastoid obliteration and the group without an obliterated mastoid (*p* = 0.73). Fifteen patients had at least one second-stage procedure to prevent recurrence and/or for ossicular chain reconstruction.

## Discussion

Labyrinthine fistula is regarded as the most frequent complication of otitis media with cholesteatoma [6, 11]. The management of a labyrinth fistula is challenging in itself and there are several other variables that add to that difficulty. The number of revision surgeries, the preoperative dizziness ratio, the number of damaged structures and deaf ears preoperatively show that our cohort consisted of very challenging cases and challenging fistulas. These challenging cases can be attributed to our tertiary care role. As mentioned earlier, several fistula classifications have been proposed for postoperative BCT. We believe that the simplicity of the Sanna classification [7] makes it a more suitable classification system for the clinic than those of Palva et al. [8], Dornhoffer et al. [2] or Quaranta et al. [13]. The different grades are easily understandable and measurable on HRCT scans or intraoperatively.

The reported incidence of fistulas in our cohort was 7%, which concurs with the literature [1–4]. In addition, the incidence of LSC fistulas only (73%) confirmed earlier publications [14, 15]. Our reported incidence of cochlea fistula (11%) was far higher than the incidence reported in the literature (<1%) [15]. This could be attributable to our position as a tertiary care referral centre. Changes in BCT due to a labyrinthine fistula vary in other publications. Sheey et al. [16] reported that 12% of patients were deaf preoperatively. Ritter [17] found a rate of 30%. In the current cohort 11 patients (24%) were deaf preoperatively, and 91% of those patients were found to have a large fistula according to the classification of Sanna et al. [7] These results suggest that there could be a correlation between fistula size and preoperative hearing. This correlation would imply that a larger fistula is linked to a higher probability of preoperative deafness. The percentages reported previously for the postoperative deterioration of BCT due to the complete removal of a cholesteatoma from the fistula vary between 0 and 66% and the rate of deaf ears postoperatively can rise to 12% [3]. In our study, these rates were 7 and 7%, respectively. Like Jang et al. [10], Meyer et al. [18] and Sone et al. [19], we found no correlation between fistula size and postoperative BCT. Ikeda et al. [20] did find a correlation between fistula size and poorer postoperative BCT. Moon et al. [21] postulated a possible explanation for this difference: it could be that, in some studies, all patients with large fistulas were already deaf preoperatively. The retrospective evaluation of our cohort did not identify any differences between primary/revision surgery or surgical techniques in terms of postoperative hearing. Opening the membranous labyrinth during surgery involves a major risk of labyrinthine damage because intramembranous fluid can leak out. As mentioned before, this can also have an adverse effect on the cochlea. This may be attributed to an inflammatory reaction in the intralabyrinthine and intracochlear system. The fact that all labyrinthine structures are connected to each other could explain the absence of a correlation between the number of damaged structures and specific damaged structures on the one hand and postoperative BCT on the other.

As the literature assumes that intravenous corticosteroids during the peeling of the cholesteatoma matrix of the labyrinth may be beneficial [2, 10, 11, 18], we looked at this method in the light of our data. No statistical difference was found in literature search or in our direct comparison to the data of Jang et al. [10]. Mastoid obliteration can be a safe and elegant way to prevent recurrent cholesteatoma entering the mastoid cavity (Geerse et al. [22]). Jang et al. [10] stated that mastoid obliteration can also prevent pressure changes from the ear canal and therefore prevent postoperative dizziness. We did not find this in our cohort, possibly because our method of ‘fistula sealing’ already protects the membranous labyrinth from pressure changes. Most publications do not give details about residual or recurrent cholesteatoma. Meyer et al. [18] had a residual rate of 19%, which is twice as high as ours. On the other hand, they had only one postoperative deaf ear. As stated previously, we believe that there could be a correlation between the thorough eradication of the cholesteatoma matrix from the fistula and postoperative hearing. Our high rate of opened membranous labyrinth was linked to a low residual rate, but the finding of two deaf ears postoperatively also supports our hypothesis, the appraisal of which could benefit from residual rates from other studies. Both variables should be taken into account to arrive at fair results. It is remarkable that no residual cholesteatoma was found in ears which were partially obliterated, although obliteration was performed in almost two-thirds of the cohort. A possible explanation could be that obliteration also prevents any remaining cholesteatoma matrix from developing into a genuine residual cholesteatoma. This finding could be an additional argument in favour of a single-stage surgical procedure. Hinohira et al. [23] found less epithelia growth in obliterated cavities in an animal model. Since this was an animal model that used tissue other than real cholesteatoma, we believe more clinical data are needed to support this hypothesis.

## Conclusion

This study shows that labyrinthine fistulas are mostly located in the LSC. Large fistulas are correlated with deteriorated preoperative BCT, but not with deteriorated postoperative BCT. Fistula size is therefore not clinically useful as a predictive parameter during counselling. Opening the membranous labyrinth during surgery is correlated with deteriorated postoperative BCT and can be seen as a predictive parameter in this type of surgery. The present study and the literature have found no protective effect relating to postoperative BCT associated with the use of corticosteroids in the surgical management of labyrinthine fistula. Despite the fact that no conclusions can be drawn about the balance between the eradication of cholesteatoma matrix from the labyrinthine fistula and postoperative hearing, we propose the more widespread publication of both variables to establish an accurate and fair picture of the results in combination.
